# Synergistic effect of drought and rainfall events of different patterns on watershed systems

**DOI:** 10.1038/s41598-021-97574-z

**Published:** 2021-09-23

**Authors:** Jiali Qiu, Zhenyao Shen, Guoyong Leng, Guoyuan Wei

**Affiliations:** 1 State Key Laboratory of Water Environment Simulation, School of Environment, Beijing Normal University, Beijing, 100875 People’s Republic of China; 2 Key Laboratory of Water Cycle and Related Land Surface Processes, Institute of Geographic Sciences and Natural Resources Research, Chinese Academy of Sciences, Beijing, 100101 People’s Republic of China

**Keywords:** Environmental sciences, Hydrology

## Abstract

The increase in extreme climate events such as flooding and droughts predicted by the general circulation models (GCMs) is expected to significantly affect hydrological processes, erosive dynamics, and their associated nonpoint source (NPS) pollution, resulting in a major challenge to water availability for human life and ecosystems. Using the Hydrological Simulation Program–Fortran model, we evaluated the synergistic effects of droughts and rainfall events on hydrology and water quality in an upstream catchment of the Miyun Reservoir based on the outputs of five GCMs. It showed substantial increases in air temperature, precipitation intensity, frequency of heavy rains and rainstorms, and drought duration, as well as sediment and nutrient loads in the RCP 8.5 scenario. Sustained droughts followed by intense precipitation could cause complex interactions and mobilize accumulated sediment, nutrients and other pollutants into surface water that pose substantial risks to the drinking water security, with the comprehensive effects of soil water content, antecedent drought duration, precipitation amount and intensity, and other climate characteristics, although the effects varied greatly under different rainfall patterns. The Methods and findings of this study evidence the synergistic impacts of droughts and heavy rainfall on watershed system and the significant effects of initial soil moisture conditions on water quantity and quality, and help to guide a robust adaptive management system for future drinking water supply.

## Introduction

Changing climate in recent decades has significantly increased extreme events at global scale. Some 27 indices for climate extremes have been recommended by the ETCCDMI (http://etccdi.pacificclimate.org/list_27_indices.shtml), including percentile-based, absolute, threshold, duration, and other indices related to changes in intensity, frequency, and duration of precipitation and temperature events, such as number of heavy rainfall or rainstorm days and periods of excessive wetness, dryness, warmth, or cold^1^. Precipitation shifts alter the volume and timing of storm and snowmelt runoff into water bodies, which has triggered hazards with increasing soil erosion and pollutant export in watersheds^2,3^. Rising temperatures increase both evaporative and transpiration losses from land and water surfaces, potentially reducing annual runoff and streamflow, which will present a challenge of insufficient water-supply capacity to meet water demands for ecosystem, agriculture, domestic, and industrial use. Furthermore, warmer air can trigger more rainfall extremes because it is associated with more atmospheric moisture and local convective events that provide more potential energy to drive rainstorms^4^. The frequency of long-term low rainfall is predicted to increase by the end of the twenty-first century^5,6^. Flash flood hazards and rainfall’s erosive power will increase if an increase in precipitation is accompanied by increases in drought duration and rainfall intensity, and nutrient losses and pollutant export will be further aggravated.

Average temperature and precipitation are not the only factors that affect watershed systems. Extreme climate conditions such as sustained droughts, extreme flooding, and intense rainfall events have major effects on hydrology and water quality^7,8^. Extreme dry and wet events may increase decomposition or other complex interactions and may flush more pollutants into surface water^9,10^. Extreme rainfall events can result in a variety of water quality impacts and damage water supply systems due to their vulnerability and low adaptation capacity to short-term transient events. Heavy rainfall and storms were the most common extreme weather events with prompt outbreaks, which significantly threatened the security and supply of drinking water. Furthermore, intense precipitation after a long drought period will increase soil erosion and flush more nutrient loads into streams, which will increase the risk of eutrophication in the reservoir. Therefore, high intensity precipitation events and long antecedent drought duration are the critical climate extremes threatening drinking water security and ecosystem health^11^. It is important to enhance the adaptive capacity of all watershed management systems in the face of climate variability and extremes, including robust risk management approaches and potential adaptive management strategies with Best Management Practice (BMP) configurations. However, a clear understanding of the interaction between extremes and water quality impairments is integral to climate-change mitigation and adaptation^12^.

During droughts, seasonal changes in antecedent moisture conditions, crop growth stage, agricultural practices, and climate characteristics can interactively affect surface runoff, sediment, nutrient export, and waterborne pathogens from agricultural watersheds. The impacts of drought on hydrology and water quality is highly complex, depending on the combination of weather conditions and land-surface characteristics that precede, co-occur with, or follow a long drought period^6,13–15^. Generally, the hydrological and water quality responses to a rainfall or drought event are determined by topographic, soil, and climate drivers. A systematic analysis of influencing factors incorporating the impacts of climate extremes is imperative to promote sustainable development and conservation of water resources.

Based on the downscaled outputs of general circulation models (GCMs), hydrological and watershed models have been extensively used in studies on climate variability impact^16–19^, such as the Soil and Water Assessment Tool (SWAT) model^20^, the Hydrological Simulation Program-Fortran (HSPF) model^21,22^, the Storm Water Management Model (SWMM)^23^, and the Agricultural Non-Point Source Pollution Model (AGNPS)^24^. The HSPF model is a semi-distributed, comprehensive model with an integrated framework of three modules for continuous simulation of various hydrological and associated water quality processes from pervious land areas (PERLND), impervious land areas (IMPLND), and stream channels (RCHRES) in daily and sub-daily time series^25^. It has been demonstrated to be an effective tool to evaluate short and long-term point and nonpoint source (NPS) pollution and the degradation and transport of chemical contaminants across watershed and regional scales, along with the integrated simulation of land-soil runoff processes^22,26^. Considering that extreme climate events occur over very short periods of several hours or days, the HSPF model was selected from existing watershed models due to its ability to run at sub-daily scales. Several modeling studies have also indicated that the HSPF is a reliable tool to address the effects of BMPs within watersheds under various climate change scenarios^27,28^, which is critical in developing adaptation strategies for climate extremes. With this in mind, this study used the HSPF model to reveal the impacts of climate extremes on watershed hydrology and water quality.

The Miyun Reservoir is the most important surface drinking water source for China’s capital, Beijing, providing domestic, industrial, and commercial water for a large population of 21.75 million. The drainage watershed of the Miyun Reservoir is managed with the aims of sustainably supplying clean water and improving water quality. Changes in water balance and water quality in the upstream catchment would directly challenge water supply and rapid economic development in Beijing. Generally, a watershed is more vulnerable to extreme events because of the transient devastating effects of rapid watershed processes on the water resource and the lack of retention time and the limited space for pollutant dilution or decomposition^29^. Hence, an upstream catchment of the Miyun reservoir was selected to perform a reliable evaluation of the impacts of extreme climate event on hydrology, NPS pollution and to analyze the effects of interacting factors along with climate variability. The main aim of this study was to seek hydrological and water quality responses to extreme climate (heavy precipitation and drought) on the basis of assessing the impacts of interacting factors on hydrology and NPS pollution. Therefore, the objective of this study can be achieved by completing the following tasks: 1) evaluate the variability of extreme climate in the future, including number of heavy precipitation days ≥ 25 mm, number of rainstorm days ≥ 50 mm, and periods of excessive dryness; 2) assess the potential impacts of extreme precipitation on watershed hydrology and NPS pollution; 3) assess the potential impacts of drought on watershed hydrology and NPS pollution; 4) analyze the interacting factors for watershed hydrology and water quality in response to drought conditions. The results evidenced the substantial impacts of climate changes or extremes on watershed systems, and highlighted the significant effects of initial soil moisture conditions on water quantity and quality.

## Methods

**Figure 1:**
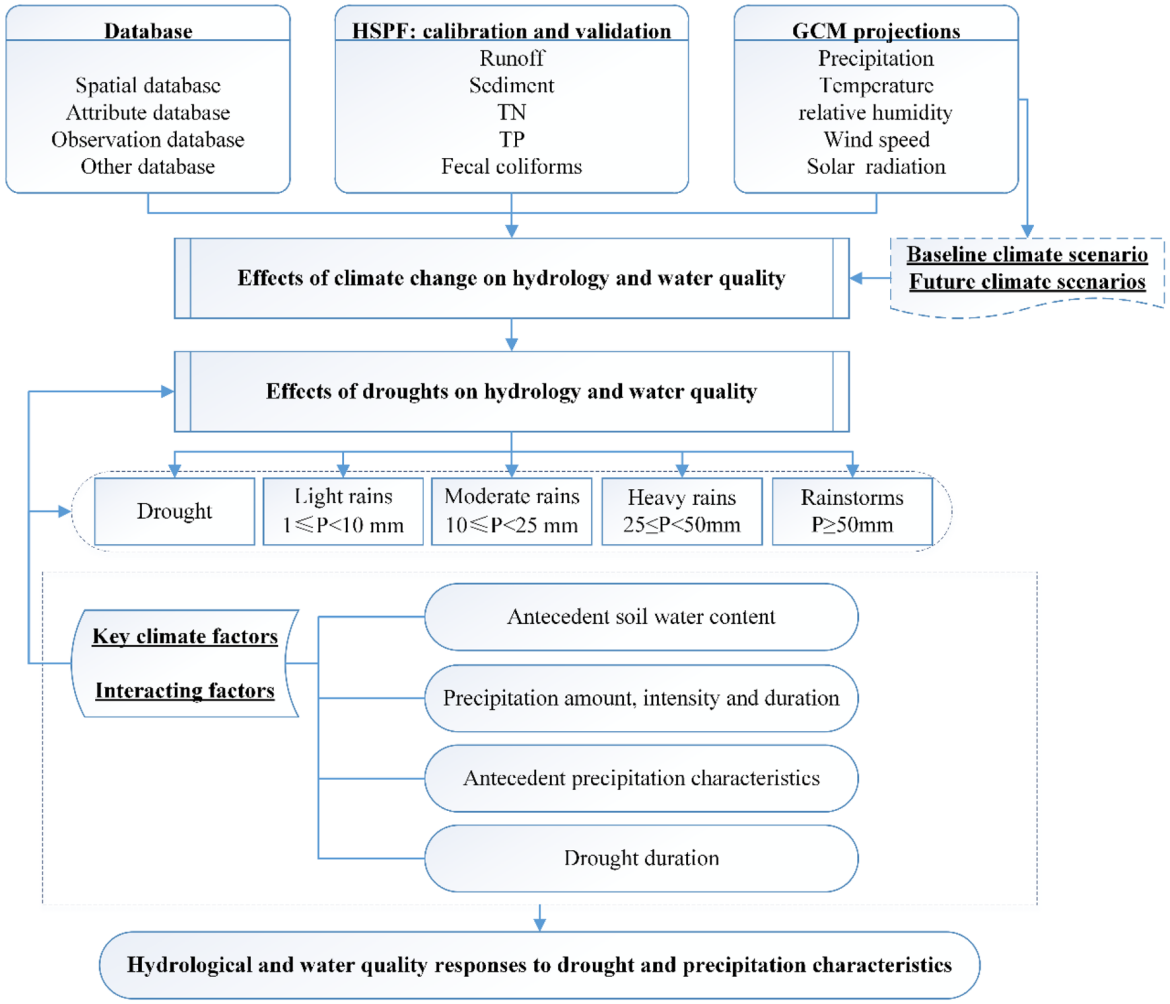
The framework for seeking potential impacts of extreme precipitation and drought on watershed hydrology and NPS pollution.

The framework for seeking potential impacts of extreme precipitation and drought on watershed hydrology and NPS pollution and identifying interacting factors is shown in Fig. 1. It included the following steps: (1) performing model setup, calibration and validation driven by the input datasets; (2) selecting, collecting and preparing GCM data, and dividing time periods for baseline and future climate scenarios; (3) identifying projected changes in precipitation and temperature, especially maximum consecutive drought days and frequency of different rainfall patterns; (4) simulating hydrological and water-quality processes using the well-calibrated model based on the outputs of multiple GCMs; (5) evaluating the impacts of climate change on watershed system; and (6) identifying interacting factors in response to rainfall patterns and drought conditions using the multiple regression analysis method^30^.

### HSPF model description, setup, calibration, and validation

The HSPF model is contained in the U.S. Environmental Protection Agency (USEPA) Better Assessment Science Integrating Point and Nonpoint Sources (BASINS) decision support tool, which incorporates the Geographic Information System (GIS). The HSPF model is a conceptual, dynamic, process-based watershed model for simulating land-surface and subsurface hydrological and associated water quality processes. It has been extensively used around the world for many purposes, including watershed planning and management, flood control, water quality management (sediment, nutrients, fecal coliforms, pesticide, and other pollutants), evaluation of BMPs, and assessment of climatic regimes and land-use change impacts^31^.

This study prepared large volumes of high-resolution physical data to setup the HSPF model, including topographic data, land-use data, soil characteristics, agricultural practice information, point sources, and meteorological forcing such as hourly and/or daily time series of precipitation, maximum and minimum air temperature, relative humidity, potential evapotranspiration (PET), solar radiation, wind speed, and cloud cover. Table S1 in the Supplementary Material shows the required data for model setup. Based on information about watershed topography and land cover, the HSPF divides a watershed into three hydrologic units: pervious land, impervious land, and stream channels. The HSPF contains various equations and parameters to simulate water balance and eventual disposition as well as transport and transformation of pollutants within streams and pervious and impervious land segments^25^. Model calibration and validation were performed based on the observed data during 2014–2015, combined with an automatic calibration program (the Model-Independent Parameter Estimation (PEST) program) and the Bacterial Indicator Tool for parameter adjustment. More details of the model description, data availability, and calibration and validation of hydrological and water quality processes in the TMC (Tables S2 to S5 in the Supplementary Material) were given in a previous study by the authors^32^. The statistical metrics (coefficient of determination (*R*^2^) and Nash–Sutcliffe efficiency (*Ens*)) indicated satisfactory performance of the HSPF model in simulating streamflow and water constituents in the study watershed (Table S5 in the Supplementary Material).

### Climate scenarios

Extreme climate projections from 2020 to 2099 were obtained from five GCMs that are recommended in the Inter-Sectoral Impact Model Inter-comparison Project (ISI-MIP), including GFDL-ESM2M, HadGEM2-ES, IPSLCM5A-LR, MIROC-ESM-CHEM, and NorESM1-M (Table S6 in the Supplementary Material), based on the Coupled Model Intercomparison Project 5 (CMIP5). The scenario 8.5 representative concentration pathway (RCP 8.5) was selected because it has the highest levels of forcing and the largest probability of climate change at the end of the twenty-first century according to the development styles of population, technology, and economics^33^ to reveal potential extreme effects on watershed systems. To reduce systematic biases in models, simulated climate datasets were corrected by a distribution-based bias-correction method, which was given in Hempel et al.^34^. The probability distributions of observations and bias-corrected simulations during 1960–2005 are shown in Fig. S1 in the Supplementary Material. Although the probability distributions of precipitation remained imperfect, a substantial harmonisation was achieved. For temperature the values showed very good agreement. These results verified the applicability of GCMs.

### Application of climate change scenarios in the HSPF model

The climate projections obtained from the five GCMs were divided into in baseline period (1980–2004) and future period (2020–2099). Projected changes in annual precipitation amount, annual mean temperature, maximum temperature, minimum temperature, maximum consecutive drought days, and frequency of different rainfall patterns was identified by comparing the future climate data with the baseline climate condition. As the model was calibrated and validated, the climate projections were used as input into the HSPF model to identify the impacts of climate change on water balance and quality. Daily precipitation dataset was temporally disaggregated into an hourly time series with the support of historical observed data to provide a better accounting of the short duration of intense rainfall within the HSPF model. That is, the temporal distribution of hourly observed data is extended to that of GCM hourly data for the corresponding analog day. The disaggregated downscaled hourly precipitation is calculated as follows:

$$ P_{s,i,j} = \frac{{P_{o,i,j} }}{{P_{o,i} }}P_{s,i} $$

where $P_{s,i,j}$ is the *j*th hour in the *i*th day temporal disaggregated GCM data, $P_{o,i,j}$ is the hourly observed data of the *j*th hour in the *i*th day, $P_{o,i}$ is the daily observed data of the day *i*, and $P_{{{\text{s}},i}}$ is the daily simulated data of the day *i* from GCM. More details on this disaggregation method can be found in the Leng and Tang^35^ and Vormoor and Skaugen^36^.

Three major extreme events were considered in this study: intense precipitation events with depth greater than 25 mm but less than or equal to 50 mm, intense precipitation events with depth over 50 mm, and long-term droughts.

## Case study applications

**Figure 2:**
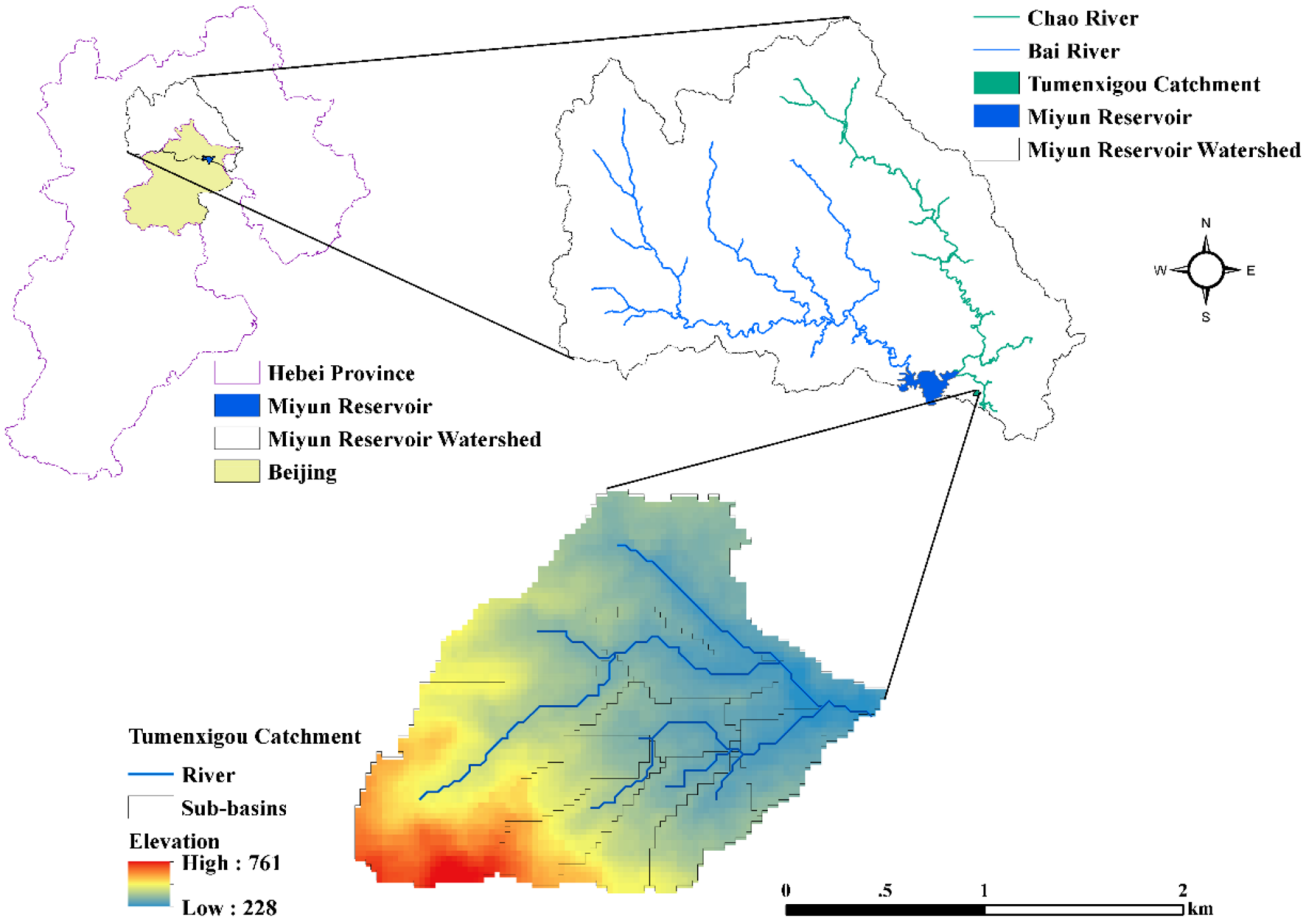
Geographical location of the study watershed. This figure was created with ArcMap 10.2 (http://www.arcgis.com/).

The Tumenxigou catchment (TMC, N40° 40′, E116° 20′, see Fig. 2) is an upstream catchment of the Miyun Reservoir. It was selected as the study area for analyzing extreme climate impacts on hydrology and water quality. It lies 5 km east of the Miyun Reservoir and covers an area of 3.27 km^2^. The landforms are mainly hilly, with an elevation range of 228–761 m, and nearly half of the catchment has slopes greater than 25°. Land use in the TMC is predominantly forest (57.78%, including artificial ecological and economic forests), cropland (13.62%), dry fruit plantations or orchards (26.52%), and rural residential areas (2.07%). The climate is of semi-humid continental monsoon type, with the mean annual temperature and precipitation being 14.6 °C and 660 mm respectively. Because point sources are rare, pollutants are exported mainly from diffuse sources and flow naturally to the outlet. As an upstream catchment of the Miyun Reservoir, the water quality of the TMC should be improved, especially during rainfall events, with strict permissible limits that are compliant with the Class III Environmental Quality Standards for Surface Water in China (GB3838-2002, see Table S7 in the Supplementary Material)^32^.

### Projected changes in precipitation and temperature

In the TMC, the projected annual precipitation increased by 3.69% to 13.50% over three future periods compared to the baseline climate conditions, with the largest increase in the middle century period and the lowest in the early century period (Table 1). Mean annual temperature increased by 1.53 °C to 3.65 °C relative to the baseline period, with the largest increase in the late century. Annual maximum temperatures were projected to increase by 1.21 °C to 4.17 °C, with the largest increase in the middle century, whereas annual minimum temperatures increased by 3.02 °C to 3.52 °C, with the largest increase in the late century.

**Table 1:** Average annual change in precipitation, mean temperature, daily maximum temperature, and daily minimum temperature across five GCMs in three future periods related to the baseline period in the TMC.

Variable	Baseline	Average annual change		
		Early century (2020–2044)	Middle century (2045–2069)	Late century (2070–2099)
Precipitation	598.33 mm	22.08 mm (3.69%)	46.54 mm (7.78%)	80.79 mm (13.50%)
Mean temperature	12.22 °C	1.53 °C (12.52%)	2.59 °C (21.19%)	3.65 °C (29.87%)
Maximum temperature	38.57 °C	1.21 °C (3.14%)	4.17 °C (10.81%)	3.95 °C (10.24%)
Minimum temperature	− 15.70 °C	3.02 °C (19.23%)	3.26 °C (20.76%)	3.52 °C (22.42%)

**Figure 3:**
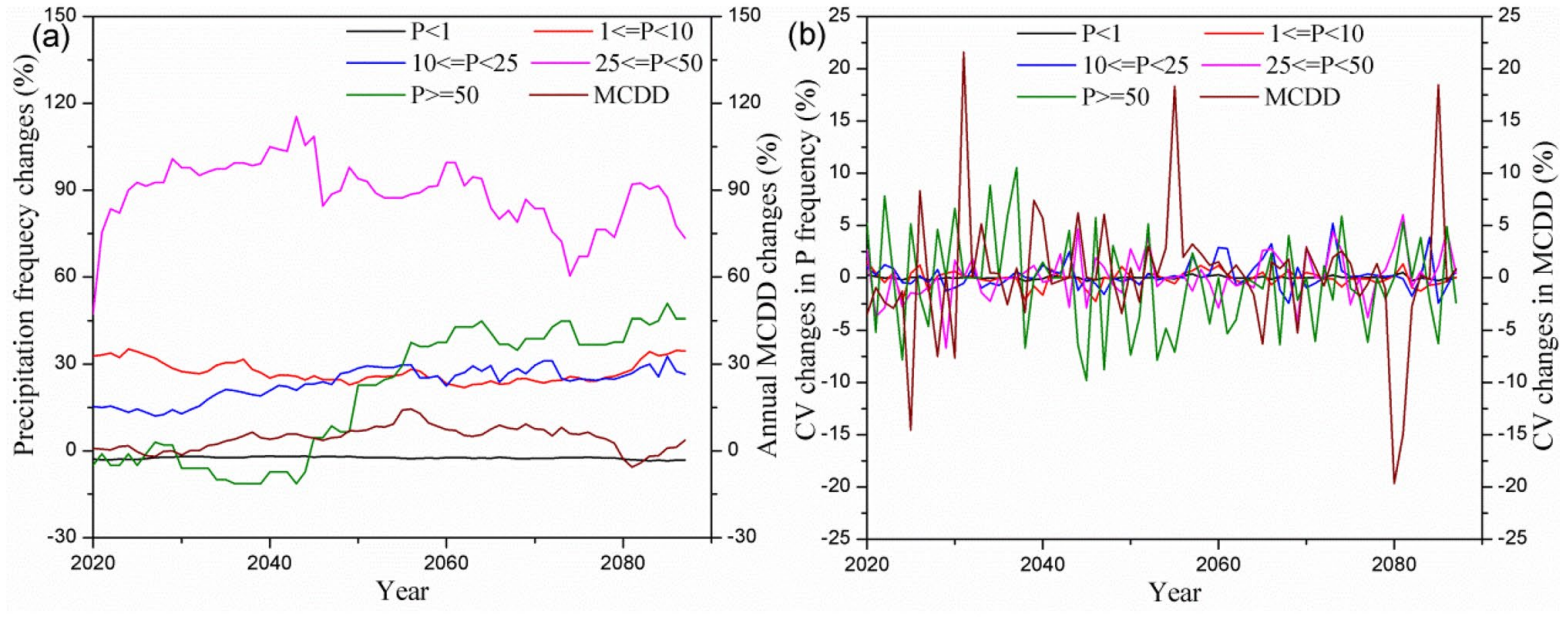
Ensemble average percentage changes in maximum consecutive drought days (MCDD) and precipitation frequency of different rainfall patterns (**a**) and their CV values (**b**) throughout 2020–2099 under the RCP 8.5 scenario compared to the baseline period. The changes were calculated as running average for each 25 years corresponding to 1980–2004. Graphs were created using OriginPro software version 9.0 (https://www.originlab.com/).

The number of annual wet days with recorded precipitation more than 1 mm increased substantially in the future climate scenario. Figure 3 shows the ensemble average changes in frequency of different precipitation patterns and their CV values in a running average of 25 years related to the baseline period. According to the amount of precipitation, rainfall patterns were classified as light rain (1 ≤ P < 10 mm), moderate rain (10 ≤ P < 25 mm), heavy rain (25 ≤ P < 50 mm) and rainstorms (P ≥ 50 mm). The data clearly show a significant increase in rainfall patterns of 25 ≤ P < 50 mm and P ≥ 50 mm, indicating a substantial increase in precipitation intensity (Fig. 3a). The annual rainfall frequency fluctuated greatly, especially the rainfall pattern of P ≥ 50 mm, followed by 25 ≤ P < 50 mm and 10 ≤ P < 25 mm (Fig. 3b), which highlighted the variability of precipitation amount and patterns in the future, which can be expected to alter the magnitude and frequency of soil erosion and nutrient export and to contribute to loss of ecosystem services in watersheds. Compared to normal conditions, intensive precipitation events with projected increases in frequency, severity, and duration had more substantial effects on watershed hydrology processes and increased peak concentrations of pollutants and pathogenic bacteria through runoff, washoff, soil erosion, and raw sewage overflows, thus leading to serious impacts on drinking-water quality in the Miyun Reservoir. Similar observations were reported by other studies^6,13,37,38^. Figure 3 also shows an increase in annual maximum consecutive drought days in 2020–2099 and a large fluctuation in CV changes for a running average of 25 years in the TMC.

### Effects of climate change on hydrology and water quality

Table 2 shows the comparison of average annual precipitation, runoff, ET and sediment and nutrient loads between baseline scenario and future climate scenarios throughout 2020–2099 obtained from five GCMs driven by RCP 8.5. The average annual precipitation in future period increased by 8.32% compared to baseline scenario. A decrease of 9.23% in annual runoff was caused by a large increase in ET (9.61%) due to significantly increasing temperatures noted in Table 1; this result was supported in previous studies^39,40^. A decrease in natural runoff to rivers or streams will contribute to more insufficient water-supply capacity of the Miyun Reservoir. Sediment loads were substantially increased by 10.95% under future climate change, which was consistent with the increases of precipitation amount and intensity noted in Fig. 3. Rainfall-runoff is generated rapidly with large velocity once a heavy rain or rainstorm event occurred with high intensity and depth, which causes a large number of sediment particles to be washed away and leached into streams in the soil erosion, exfoliation and eluviations processes, combining the hydrodynamic and erosive power of raindrops^41,42^. The average change rates of annual TN and TP load across all GCMs in the future period were 8.95% and 1.06%, respectively, compares to baseline scenario. Nutrients are dissolved in runoff or adsorbed on soil particles along with peeling, transport, deposition and suspension by the intense raindrops and surface runoff. In addition to the substantial effects of increasing precipitation and temperature proposed in our previous study^40^, an increase in the frequency of dry and heavy rainfall events in the future (Fig. 3) may be another important factor that increases the flushing, dissolution, decomposition and transport of nutrients to runoff and results in sudden outbreaks of eutrophication in water bodies^43–45^. Projected increases in the frequency and severity of extreme events and precipitant amount can result in great losses of sediment and nutrients and associated damages to drinking water supplies, which will be analyzed in more detail in the following sections.

**Table 2:** Comparison of average annual hydrology and water quality components in the TMC among baseline scenario (1980–2004) and future climate scenarios throughout 2020–2099 obtained from five GCMs driven by RCP 8.5.

Climate scenario	Water balance indicators (mm)			Water quality components		
	Precipitation	Surface runoff	ET	Sediment (t/ha)	TN (kg/ha)	TP (kg/ha)
Baseline	598.33	84.97	488.68	40.02	26.30	7.37
GFDL-ESM2M	637.92	43.15	517.42	77.94	9.43	7.86
HadGEM2-ES	711.22	66.95	555.08	72.52	45.42	8.17
IPSL-CM5A-LR	675.92	122.60	566.48	29.06	34.09	11.45
MIROC-ESM-CHEM	590.53	65.47	507.38	19.90	24.75	4.40
NorESM1-M	624.82	87.47	531.92	22.59	29.56	5.36
Average of five GCMs	648.08	77.13	535.66	44.40	28.65	7.45
% change	8.32	− 9.23	9.61	10.95	8.95	1.06

**Figure 4:**
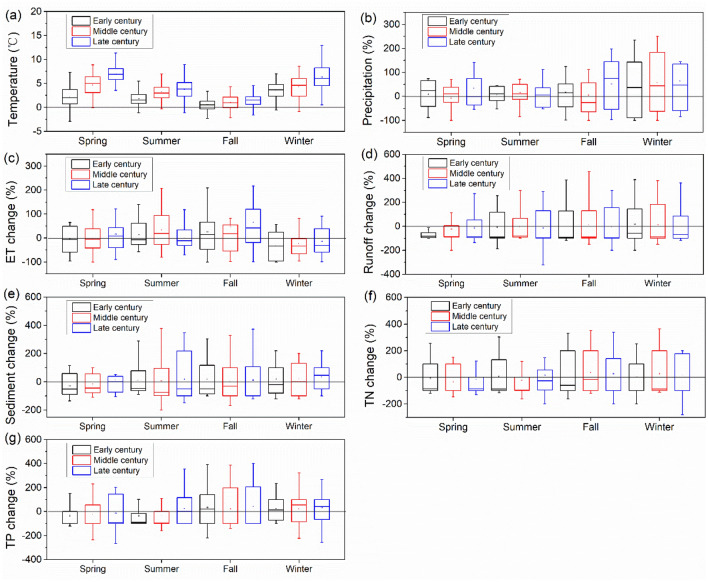
Seasonal change of temperature (**a**), precipitation (**b**), evapotranspiration (**c**), runoff (**d**), sediment (**e**), TN (**f**), and TP (**g**) on sub-basins in early, middle and late future periods related to baseline period (1980–2004). Boxes show maximum, 1st quartile, median, mean (black rectangle), 3rd quartile and minimum. Graphs were created using OriginPro software version 9.0 (https://www.originlab.com/).

Figure 4 shows the seasonal changes of precipitation, temperature, runoff, and sediment and nutrient loads for the future climates across all GCMs related to baseline climate. Precipitation and temperature showed significant seasonal variations, with low values in the dry season and peaks in the wet season, which resulted in similar seasonal variations in runoff volume, evapotranspiration (ET), sediment, and TN and TP loads, although projected results varied slightly across GCMs. Precipitation increased over the three future periods with greatest increase in winter and smallest increase in summer (Fig. 4b). Air temperature increased all the year round over the three future periods with the greatest increase in spring and winter, resulting in a great increase in ET (Fig. 4a,c). Increased temperature resulted in a decrease in runoff (Fig. 4d), especially during spring and summer, due to a substantial increase in ET, although precipitation showed an increase in the future; this result has been supported by other studies^39,46^. However, runoff increased in winter due to the increase of precipitation falling as rain than as snow along with elevated temperature during the colder period. A substantial decrease in spring was found as a result of the decrease of snowmelt runoff caused by reduced snow. Climate regimes led to great variations in pollutant export through increased rainfall intensity, runoff fluctuation and altered biochemical process. The more substantial increase in sediment load in summer and winter was driven by an increase in rainfall volume and intensity, which indicates that swift erosion of topsoil by raindrops and rainfall-runoff is the main reason for sediment loss (Fig. 4e). This finding is consistent with other future predictions^41,42,47^. An increase in nutrient export occurred with the increase in sediment load (Fig. 4f,g). Precipitation will increase in the form of rainfall instead of snowfall as temperature increases, which will contribute to a remarkable increase in nutrient loss along with an increase in runoff in winter. However, nutrient decreased in spring as runoff decreased. The increases in sediment and nutrient loads in summer and fall were primarily attributed to the increasing precipitation amount and intensity over the three future periods.

### Soil moisture responses to drought preceding rainfall events

**Figure 5:**
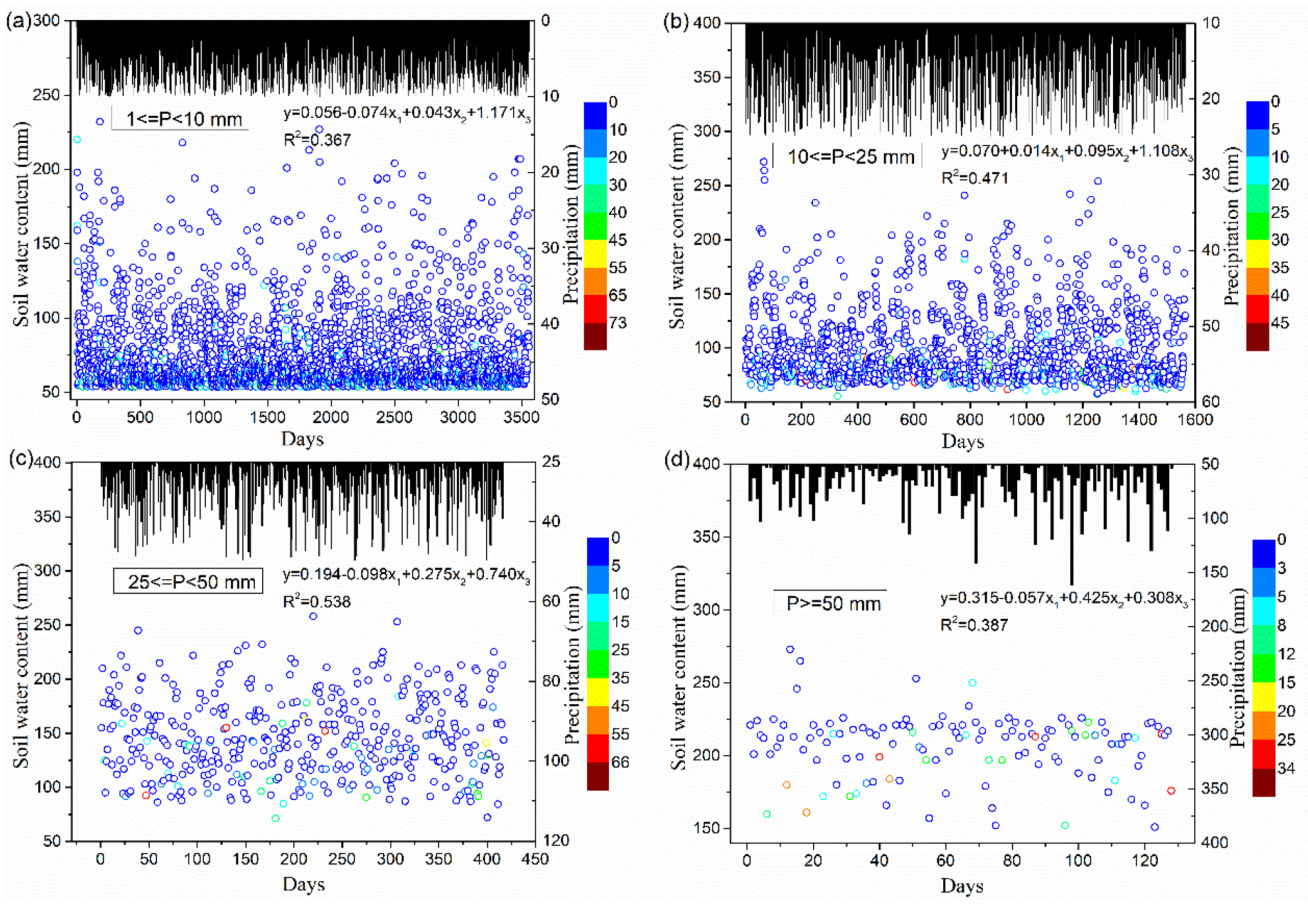
Relationship of soil water content (mm) with current and antecedent precipitation volume (mm) and antecedent dry days at different precipitation amounts: (**a**) light rain (1 ≤ P < 10 mm), (**b**) moderate rain (10 ≤ P < 25 mm), (**c**) heavy rain (25 ≤ P < 50 mm), and (**d**) rainstorm (P ≥ 50 mm). Graphs were created using OriginPro software version 9.0 (https://www.originlab.com/).

Figure 5 shows the relationships between soil moisture conditions occurring with rainfall events of different severity, which were affected by antecedent precipitation characteristics and antecedent dry days in both baseline and future scenarios. The soil moisture was analyzed using the outputs of the HSPF model. Evidently, soil water content is negatively correlated with antecedent dry days. The water exchange processe between soil, land covers, and the atmosphere during droughts is a vital component in the hydrologic cycle at the watershed scale^48^. With the increase of temperature, the increasing ET causes a large loss of soil water from soil surface to the atmosphere. Previous studies stated that the MRW had been experiencing more significant upward trend of ET than precipitation^40,49^. A great decrease in precipitation and a relatively large increase in ET on land-cover or water surfaces during a drought period substantially decreased soil moisture, interflow, baseflow, and groundwater. Thus, a high attention of drought alert should be focused on the upward trend of ET since a considerable rise in ET may also lead to a drier soil condition and reductions in surface runoff and the availability for water supply and irrigation. Root storage and soil storage are empty after a low flow period and need to be filled up again before water percolation to interflow, baseflow, and related processes can be activated, once a rainfall event occurs and the moisture in the catchment starts rising^50^. According to Fig. 5, soil water content was positively correlated with current and antecedent precipitation volume. Along with rainfall occurrence, all water infiltrates into the soil that was first held by root storage, after which it spills into soil storage to supplement soil moisture by hydraulic lift; this mechanism was supported by previous studies^51,52^. In the case of the same drought duration, more antecedent precipitation ensured a sufficient amount of soil water content before a new rainfall event occurred, although ET resulted in natural loss of soil moisture during the drought period. It can be concluded that antecedent precipitation affected soil water content primarily by affecting antecedent soil water conditions. In addition, lateral movement of soil water occurred during periods of rainfall, and larger precipitation amounts promoted more water to infiltrate into the soil, where it was held by root storage and soil storage, resulting in a systematic increase in soil water content. These results were supported by previous studies^53–55^.

Figure 5 also shows that soil water content is significantly affected by precipitation patterns. In light-rain and moderate-rain events, antecedent precipitation amount was the most important factor affecting soil water content (Fig. 5a,b). Antecedent soil water increased with greater antecedent precipitation amount, but the hydraulic gradient decreased before the catchment was wet, and hence, the infiltration rate of water decreased. The decreased infiltration rate accelerated the cumulative storage of soil water in the bottom layer, which helped to increase total soil water content. This process could continue for a longer spell in light-rain and moderate-rain events, and therefore the impact of antecedent precipitation was highlighted. The precipitation amount became a more identifiable factor in soil water content when precipitation reached a higher value as heavy rains and rainstorms (Fig. 5c,d). Because precipitation provided the main water input for soil water in natural rainstorms, a positive relationship between infiltration rates and rainfall amount and intensities was prominent. These results agree with those obtained in other studies^50,56^. Soil water content had further substantial effects on hydrological cycling and water quality in watersheds.

### Hydrological responses to drought preceding rainfall events

**Figure 6:**
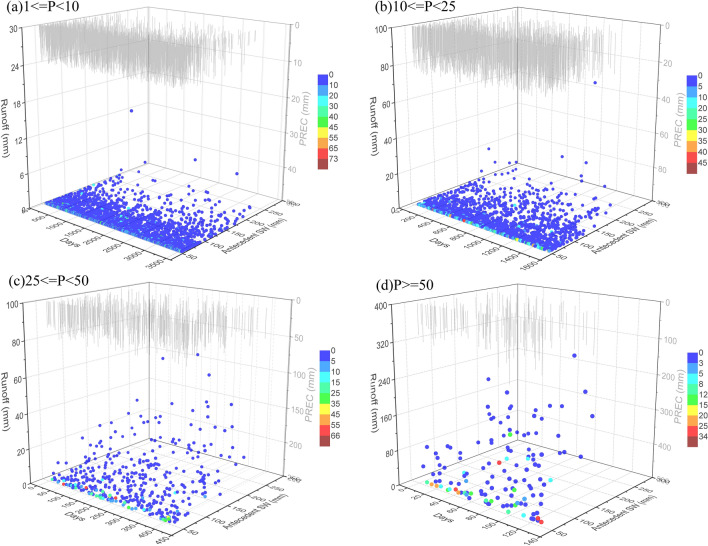
Relationship of runoff with precipitation (PREC, mm), antecedent drought days, and antecedent soil water (antecedent SW, mm) at different precipitation amounts: (**a**) light rain (1 ≤ P < 10 mm), (**b**) moderate rain (10 ≤ P < 25 mm), (**c**) heavy rain (25 ≤ P < 50 mm), and (**d**) rainstorm (P ≥ 50 mm). Graphs were created using OriginPro software version 9.0 (https://www.originlab.com/).

The hydrological response to precipitation events is determined by several interacting factors that account for runoff generation within a watershed, including initial soil conditions, topography, weather characteristics, and land cover^55,57–59^. Figure 6 shows the runoff generated under different conditions of altered precipitation amount, antecedent drought days, and antecedent soil water contents in the TCM. The results from multiple linear regression analysis (Table 3) show that antecedent soil water content played an important role in runoff generation during heavy rain and rainstorm events. The sensitivity of the runoff response to initial soil moisture depended on the predominant runoff mechanism, which was related to soil properties and precipitation characteristics. In the TMC, the predominant runoff mechanism is saturation-excess overland flow due to its semiarid environment with high permeability. When rainfall amount or soil water content is sufficiently high to meet the needs of high infiltration, watersheds produce runoff. As a result of the large amount of rainfall (Fig. 6c,d), runoff was generated when the amount of rain approached or exceeded the saturation capacity of the upper soil layer. Hence, this saturation-excess overland runoff highly depended on the degree of saturation of the upper soil layer and the initial high-permeability soil conditions within the TMC, which were consistent with the high correlation between runoff and antecedent soil water content under heavy rain and rainstorm events (Table 3). For low-amount events, the precipitation amount was far below the saturation point of the upper soil layer, and runoff was hard to generate. This can explain the low correlation between runoff and antecedent soil water content under light-rain and moderate-rain events. However, when the surface soil was near to saturation, runoff began to be generated, which is illuminated by the high-runoff points with higher antecedent soil water in Fig. 6a,b. In addition, the results from multiple linear regression analysis show that greater precipitation or antecedent precipitation amount can contribute more runoff, regardless of precipitation patterns (Table 3). Along with the increase in antecedent precipitation, antecedent soil water content increases, which is a favorable condition for runoff generation. An increase in precipitation amount, which is the main water input for watersheds, would facilitate higher water yields and more surface runoff. These results are in agreement with those given by other studies^60–63^.

**Table 3:** Relationship between runoff, ET or pollutant loads with interacting factors, including precipitation amount, antecedent drought days, antecedent precipitation, and antecedent soil water contents under different rainfall patterns (M_1_ represents the regression model with explanatory variables excluding runoff, and M_2_ represents the regression model that includes runoff as one of the explanatory variables).

Response variable	Explanatory variable	Coefficient							
		Rainfall patterns							
		Light rain (1 ≤ P < 10 mm)		Moderate rain (10 ≤ P < 25 mm)		Heavy rain (25 ≤ P < 50 mm)		Rainstorm (P ≥ 50 mm)	
		M_1_	M_2_	M_1_	M_2_	M_1_	M_2_	M_1_	M_2_
Runoff	Intercept	− 0.002 (*R*^2^ = 0.070^a^)		− 0.016 (*R*^2^ = 0.218)		− 0.1039 (*R*^2^ = 0.673)		− 0.040 (*R*^2^ = 0.791)	
	Antecedent drought days	0.008		0.030		− 0.042		− 0.032	
	Precipitation amount	0		0.011		0.275		0.708	
	Antecedent precipitation	0.082		0.155		0.106		0.074	
	Antecedent soil water content	0		0.002		0.366		0.303	
ET	Intercept	0.206 (*R*^2^ = 0.008)	0.206 (*R*^2^ = 0.007)	0.521 (*R*^2^ = 0.007)	0.520 (*R*^2^ = 0.002)	0.633 (*R*^2^ = 0.006)	0.0646 (*R*^2^ = 0.006)	0.813 (*R*^2^ = 0.025)	0.811 (*R*^2^ = 0.017)
	Antecedent drought days	− 0.020	− 0.021	0.006	0.008	− 0.005	0	− 0.063	− 0.064
	Precipitation amount	− 0.016	− 0.016	0.002	0.002	− 0.010	− 0.026	0.156	0.180
	Antecedent precipitation	− 0.059	0.053	0.102	0.108	− 0.034	− 0.060	− 0.058	− 0.044
	Antecedent soil water content	0.020	0.031	0.087	0.084	0.065	0.031	0.309	0.307
	Surface runoff	–	0.098	−	− 0.048	–	0.096	–	− 0.035
Sediment	Intercept	0 (*R*^2^ = 0.010)	0.001 (*R*^2^ = 0.735)	− 0.002 (*R*^2^ = 0.022)	0.004 (*R*^2^ = 0.151)	− 0.002 (*R*^2^ = 0.403)	0.059 (*R*^2^ = 0.503)	0.034 (*R*^2^ = 0.581)	0.075 (*R*^2^ = 0.776)
	Antecedent drought days	0	− 0.006	− 0.006	− 0.017	− 0.216	− 0.193	− 0.119	− 0.087
	Precipitation amount	0.001	0	0.016	0.013	0.387	0.230	0.676	− 0.027
	Antecedent precipitation	0.008	− 0.052	− 0.021	− 0.073	− 0.261	− 0.321	0.004	− 0.069
	Antecedent soil water content	0	0	0.008	0.002	0.134	− 0.075	0.062	− 0.239
	Surface runoff	–	0.730	–	0.335	–	0.570	–	0.992
TN	Intercept	0 (*R*^2^ = 0.007)	0.001 (*R*^2^ = 0.822)	− 0.012 (*R*^2^ = 0.111)	0.005 (*R*^2^ = 0.777)	− 0.058 (*R*^2^ = 0.380)	0.017 (*R*^2^ = 0.600)	0.001 (*R*^2^ = 0.509)	0.032 (*R*^2^ = 0.605)
	Antecedent drought days	0.002	− 0.004	0.028	− 0.006	0.012	0.043	− 0.064	− 0.040
	Precipitation amount	0.001	0	0.008	− 0.004	0.259	0.058	0.527	− 0.002
	Antecedent precipitation	0.023	− 0.041	0.177	0.006	0.215	0.137	0.225	0.170
	Antecedent soil water content	− 0.001	0	− 0.012	− 0.034	0.070	− 0.198	0.060	− 0.167
	Surface runoff	–	0.777	–	1.101	–	0.730	–	0.748
TP	Intercept	− 0.001 (*R*^2^ = 0.038)	0 (*R*^2^ = 0.946)	− 0.018 (*R*^2^ = 0.204)	0 (*R*^2^ = 0.916)	− 0.094 (*R*^2^ = 0.553)	0.001 (*R*^2^ = 0.826)	− 0.020 (*R*^2^ = 0.613)	0.013 (*R*^2^ = 0.775)
	Antecedent drought days	0.006	− 0.002	0.035	0	− 0.025	0.014	− 0.043	− 0.017
	Precipitation amount	0	0	0.014	0.002	0.295	0.042	0.479	− 0.108
	Antecedent precipitation	0.057	− 0.016	0.187	0.010	0.159	0.062	0.162	0.101
	Antecedent soil water content	− 0.002	− 0.001	0.018	− 0.005	0.261	− 0.076	0.148	− 0.103
	Surface runoff	–	0.884	–	1.145	–	0.921	–	0.828
Fecal coliform	Intercept	0 (*R*^2^ = 0.020)	0 (*R*^2^ = 0.998)	− 0.029 (*R*^2^ = 0.176)	− 0.002 (*R*^2^ = 0.986)	0.001 (*R*^2^ = 0.215)	0.001 (*R*^2^ = 0.814)	0.282 (*R*^2^ = 0.249)	0.190 (*R*^2^ = 0.319)
	Antecedent drought days	− 0.008	0.002	0.004	0	− 0.136	0.147	0.006	0.010
	Precipitation amount	0.049	− 0.002	0.366	0.005	1.092	0.188	5.346	2.828
	Antecedent precipitation	− 0.005	0	− 0.035	− 0.001	0.001	0.001	− 0.264	− 0.186
	Antecedent soil water content	− 0.008	0	− 0.016	− 0.003	− 0.024	− 0.027	− 0.272	− 0.171
	Surface runoff	–	1.126	–	5.893	–	0.848	–	0.560

^a^*R*^2^ represents the coefficient of determination of the regression models with significance level *p* < 0.05.

Because the antecedent soil water content was significantly affected by antecedent drought days, runoff response to antecedent drought days should be identified. According to the results from multiple linear regression analysis (Table 3), runoff was negatively correlated with antecedent drought days under heavy rain and rainstorm events, whereas there was a positive correlation under light rain and moderate rain events. According to other studies^64–66^, soil hydrophobicity has been found to be caused by prolonged drought periods, which causes soil pores lined with organic materials or mineral oxides to exacerbate their water repellence and substantially reduces the affinity of soil for water, making surface runoff easy to generate. However, during heavy rain and rainstorm events, runoff generation was predetermined by precipitation amount, but was less sensitive to soil hydrophobicity. Longer antecedent drought duration caused low antecedent soil water content, which further decreased saturation-excess overland flow.

**Figure 7:**
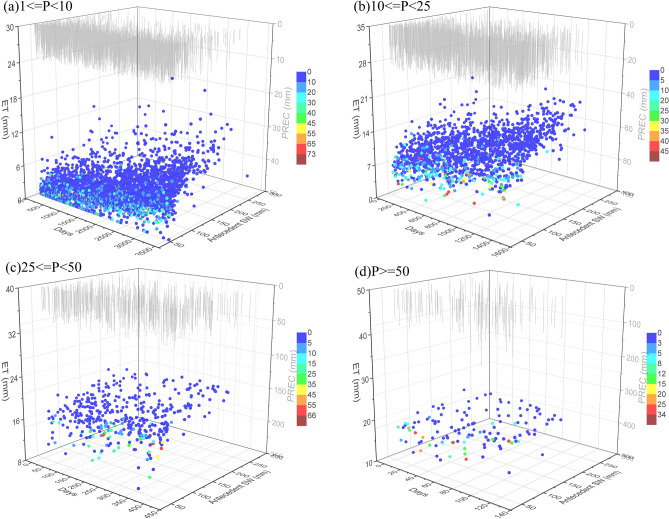
Relationship of ET with precipitation (PREC, mm), antecedent drought days, and antecedent soil water (antecedent SW, mm) at different precipitation amounts: (**a**) light rain (1 ≤ P < 10 mm), (**b**) moderate rain (10 ≤ P < 25 mm), (**c**) heavy rain (25 ≤ P < 50 mm), and (**d**) rainstorm (P ≥ 50 mm). Graphs were created using OriginPro software version 9.0 (https://www.originlab.com/).

ET is the link between the surface water and energy balances with plant physiological activity and is usually controlled by several factors, such as soil moisture, vegetation cover, and meteorological conditions^67^. Figure 7 shows the ET under different conditions of altered precipitation amount, antecedent drought days, and antecedent soil water contents in the TMC. The results from multiple linear regression analysis show a greater effect of antecedent soil water content on ET. Soil moisture storage plays an important role in the land–atmosphere water transport system in the TMC. Soil moisture dynamics are mainly controlled by rapid infiltration and subsequent vertical redistribution in the root zone, which is highly related to water uptake by roots for ET during rainfall periods^68^. Similar observations were reported by other studies^68,69^. Brandes and Wilcox^69^ found that ET had a distinctly bimodal annual pattern that coincide with high soil moisture. Additionally, during rainstorm events, ET was also predetermined by precipitation amount. The larger precipitation can stimulate more ET, indicating that rainwater stored in the soil profile could be an important part of the water budget of land cover and the hydrological cycle in the TMC. Similar observations were reported by other studies^70–72^.

### Effects of drought on soil erosion

**Figure 8:**
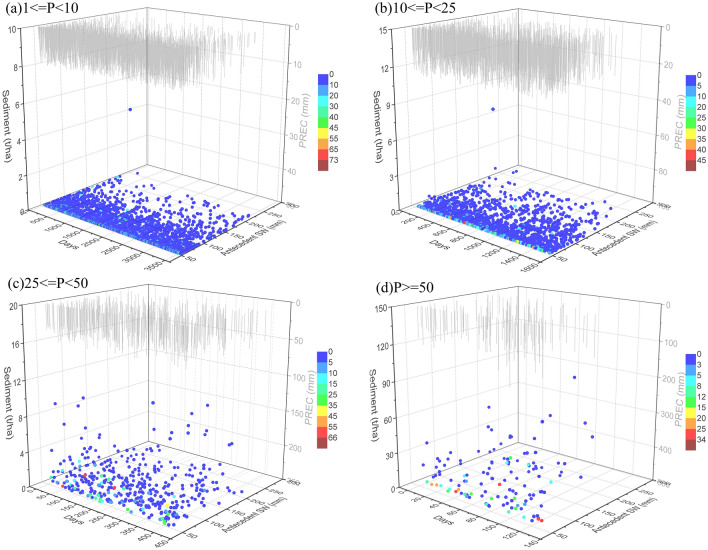
Relationship of sediment load with precipitation (PREC, mm), antecedent drought days, and antecedent soil water (antecedent SW, mm) at different precipitation amounts: (**a**) light rain (1 ≤ P < 10 mm), (**b**) moderate rain (10 ≤ P < 25 mm), (**c**) heavy rain (25 ≤ P < 50 mm), and (**d**) rainstorm (P ≥ 50 mm). Graphs were created using OriginPro software version 9.0 (https://www.originlab.com/).

The negative consequences of drought stress on watersheds can be exacerbated by increasing water contamination because rainfall-runoff following prolonged periods of drought can suddenly mobilize accumulated pollutants into streams or water bodies. Pollutant concentrations may remain elevated for a considerable period of time, despite an initial decrease in pollutant concentrations during droughts. Figure 8 shows the sediment load under interacting conditions of altered precipitation amount, antecedent drought days, and antecedent soil water contents at different rainfall patterns in the TCM. High sediment load occurred along with rainstorm events that led to rapid yields, up to 16.5 t/ha on average. Sheet and rill erosion caused by the interactive effects of raindrop action and surface runoff was the main process of sediment export, which was determined by the predominant runoff mechanism related to initial soil conditions and precipitation amount. This is consistent with other work suggesting the substantial influence of the erosive forces of surface runoff on sediment yields within watersheds^47,73^. Consequently, the sediment load was positively correlated with the precipitation amount of the current event. However, it showed a negative correlation with antecedent precipitation because a larger amount of antecedent rainfall could reduce accumulated sediment before the current rainfall event.

Antecedent soil water content had more significant effects on sediment export under heavy rain and rainstorm events than under light rain and moderate rain events, a conclusion supported by the results of multiple linear regression analysis in Table 3. An increase in antecedent soil water content increases the resistance of an aggregate to the forces of raindrop and runoff, resulting in a decrease in soil erosion rates. This result is consistent with other studies^74,75^. Soil erosion caused by runoff was the main process of sediment export, which was determined by the predominant runoff mechanism related to initial soil conditions and precipitation amount. Consequently, the sediment load was positively correlated with precipitation amount. However, it showed a negative correlation with antecedent precipitation because a larger amount of antecedent rainfall could reduce sediment accumulation before the current rainfall event.

### Effect of drought on nutrient loss

**Figure 9:**
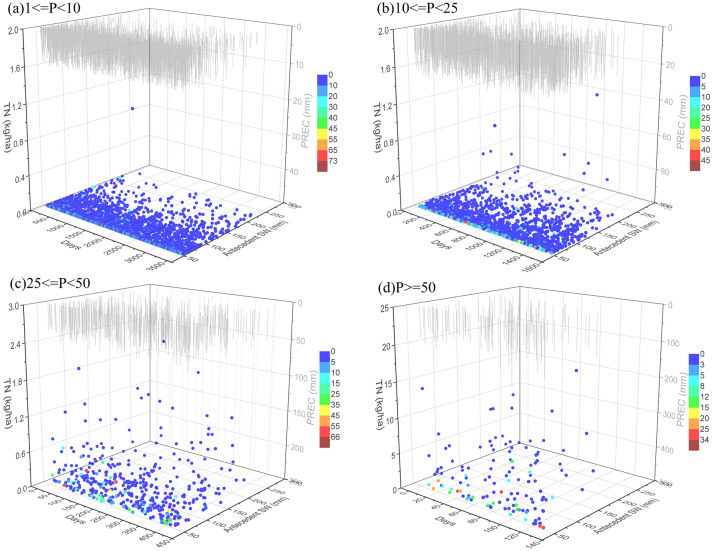
Relationship of TN load with precipitation (PREC, mm), antecedent drought days, and antecedent soil water (antecedent SW, mm) at different precipitation amounts: (**a**) light rain (1 ≤ P < 10 mm), (**b**) moderate rain (10 ≤ P < 25 mm), (**c**) heavy rain (25 ≤ P < 50 mm), and (**d**) rainstorm (P ≥ 50 mm). Graphs were created using OriginPro software version 9.0 (https://www.originlab.com/).

**Figure 10:**
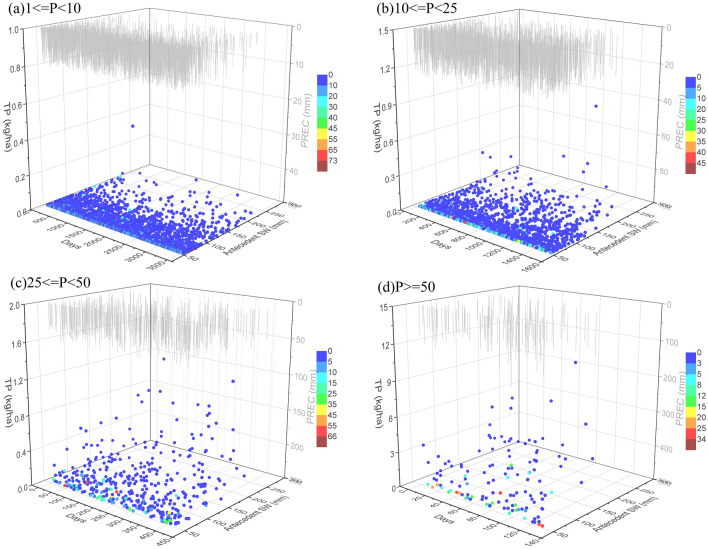
Relationship of TP load with precipitation (PREC, mm), antecedent drought days, and antecedent soil water (antecedent SW, mm) at different precipitation amounts: (**a**) light rain (1 ≤ P < 10 mm), (**b**) moderate rain (10 ≤ P < 25 mm), (**c**) heavy rain (25 ≤ P < 50 mm), and (**d**) rainstorm (P ≥ 50 mm). Graphs were created using OriginPro software version 9.0 (https://www.originlab.com/).

The relationships of TN and TP loads with interacting factors under different precipitation patterns (Figs. 9, 10, Table 3) were significantly similar to that of sediment export. This is probably the good correlation of nutrient and sediment considering the great quantity of nutrients sorbed to the soil or to soil organic matter along with soil erosion. This result was in agreement with those given by other studies^60,76^. As shown in Figs. 9 and 10, heavy rainfall and rainstorm events increased mass export of nutrients. According to the results of multiple linear regression analysis in Table 3, strong responses of nutrient export to rainfall and runoff were identified. If the impact of runoff were excluded, the precipitation amounts of current and antecedent events were the key factors influencing nutrient loss, which were the primary factors driving runoff generation and could influence the “first flush” of nutrient concentrations during rainfall events. Land surface is exposed to the direct impact of millions of raindrops and surface runoff, which can result in high soil erosion and associated nutrient loss rates through dislodging, splashing, and scouring of soil particles and washing out of soil nutrients into streams and water bodies. These results agreed with those in other studies^13,32^. However, an increase in antecedent soil water content resulted in a decrease in nutrient export, especially under heavy rain and rainstorm events. This is in effect a reduction in sediment export as a result of increasing antecedent water content. Meanwhile, nutrient loss could be reduced from rainfall events, considering the driving effect of soil erosion on nutrient loss. In addition, nutrient load was found to be proportional to the number of drought days. Increased drought duration can lead to more accumulation of nutrients on land surfaces, from which they are rapidly flushed to streams during rainfall events.

### Effect of drought on fecal coliforms

**Figure 11:**
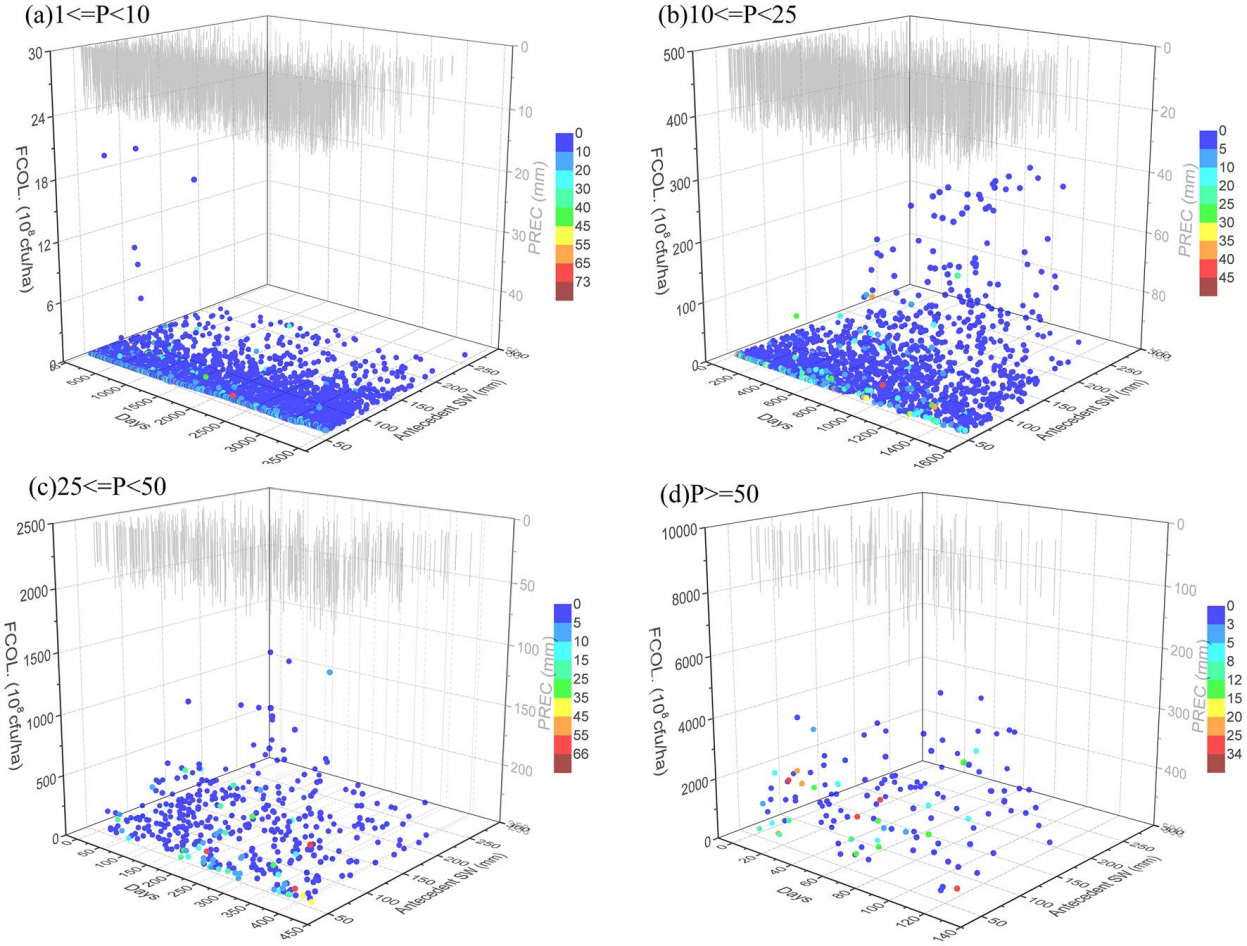
Relationship of fecal coliforms with precipitation (PREC, mm), antecedent drought days, and antecedent soil water (antecedent SW, mm) at different precipitation amounts: (**a**) light rain (1 ≤ P < 10 mm), (**b**) moderate rain (10 ≤ P < 25 mm), (**c**) heavy rain (25 ≤ P < 50 mm), and (**d**) rainstorm (P ≥ 50 mm). Graphs were created using OriginPro software version 9.0 (https://www.originlab.com/).

Because fecal coliform contamination, which is a driver of health outcomes, has been a major threat to global drinking water safety, many studies have focused on quantifying loads or concentrations over a range of hydrologic conditions and identifying their sources^38,77^. Fecal contamination has been proven to be seasonal and more frequent during rainy periods^32,78^. According to the results of multiple linear regression analysis in Table 3, runoff magnitude appeared to be the best predictor of fecal coliforms, with a high coefficient of determination (*R*^2^) of 0.998. This suggests that rainfall-runoff is the major factor influencing fecal coliform contamination. Runoff scours topsoil that incorporates intensive manure applications and the waste produced by animals. However, surface runoff and soil loss for individual rainfall events vary widely, depending on antecedent soil moisture conditions such as soil storage and infiltration capacity, precipitation amount, and other factors. This study found that antecedent soil water content was significantly related to runoff generation and soil erosion. An increase in antecedent soil water content resulted in a decrease in fecal coliform export, which indicated that this wet soil had low susceptibility to erosion by raindrops and runoff due to its aggregate stability and compaction. Precipitation amount played an important role in fecal coliform export because precipitation inputs directly correlated with runoff magnitude were found to be the key contributor to fecal coliform loading. After a large rainfall event, less fecal coliform is left on the land surface to be transported to streams, and hence the load was negatively correlated with antecedent precipitation amount (Table 3). Figure 11 shows that fecal coliforms occur in significantly greater loads following rainfall, especially heavy rainfall and rainstorm events that have been preceded by drought. Longer drought duration typically means a higher accumulation rate of fecal coliform on the land surface, contributing to a higher load from the catchment. These results agreed with those shown in Table 3, which indicated a positive correlation between fecal coliform loads and antecedent drought days.

Generally, the average concentrations of TN, TP, and fecal coliform bacteria in all heavy rains in the period under study respectively reached 1.63 mg/L, 0.225 mg/L, and 17,154 cfu/L at the outlet of the TCM, which exceeded the limits of the Class III Environmental Quality Standards for Surface Water by 63.31%, 12.53%, and 71.54% respectively. Furthermore, on average during all rainstorm events, TN, TP, and fecal coliform concentrations respectively reached 1.74 mg/L, 0.229 mg/L, and 18,375 cfu/L, which exceeded the limits by 74.25%, 14.54%, and 83.75% respectively. Heavy rain and storm runoff evidently caused serious contamination of surface water bodies. Furthermore, long antecedent drought duration consequently led to more serious impacts on drinking water quality and ecosystem services in the Miyun Reservoir. Therefore, the development of watershed management strategies for adapting to climate change should focus on the rainfall-runoff processes of these two precipitation patterns after prolonged drought.

### Uncertainty and limitations of assessment method

The climate data for both temperature and precipitation used in the current impact analysis may not be accurate at watershed scale due to the uncertainties arising from multiple sources such as GCM structure, emission scenarios, and downscaling or bias-correction methods^79–82^. More confidence in climate change impact studies will be expected from projections based on both GCMs and regional climate models (RCMs). GFDL-ESM2M, HadGEM2-ES, IPSLCM5A-LR, MIROC-ESM-CHEM, and NorESM1-M are recommended in the framework of ISI-MIP, which aims to synthesize climate change impacts on water resources, agriculture and ecosystems in different sectors and at different scales, and to identify the inherent uncertainty in climate models and impact models^83–85^. Within the ISI-MIP, only these five GCMs from the CMIP5 driven by multiple RCP scenarios were bias corrected to provide climate forcing for watershed models, which makes it impossible to fully characterize the potential uncertainty ranges by GCMs. However, a greater fraction of the ensemble range coverage can be captured by the five GCMs in the ISI-MIP framework than any other 500 subsets of five GCMs from CMIP5, with median 0.75 and 0.59 across different regions and seasons for temperature and precipitation, respectively, which better represents the range of changes in regional climate^86^. Numerous previous studies have reported the applicability of climate projections from these five GCMs as input data of watershed models to quantify the impact of climate change on water resources at regional scale^87–89^. Although uncertainties are inevitably introduced in the simulated results, we found that all five climate models projected the same changing pattern with different magnitudes in precipitation and temperature (Figs. S1, S2 in the Supplementary Material), suggesting good reliability in the projections with some acknowledgment of the uncertainty in ensemble outputs.

Another potential source of uncertainty is impact models, which can be attributed to model structure and parameters, skill of the modeler, and other inputs required for the simulations. The uncertainty in the input data, including measurement inaccuracy as well as random or systematic errors relating to data management, which is passed on to the simulated results in a form of aleatory uncertainty, can be calculated by generally applicable methods, such as Monte Carlo sampling method, Generalized Likelihood Uncertainty Estimation Method, and Markov Chain Monte Carlo Method^79,90^. The uncertainty in model parameters was analyzed in our previous study by parameter sensitivity analysis and model calibration using the PEST program to improve the fit between simulated and observed data^32^. Furthermore, another important source of uncertainty occur in the modelling process is the errors related to incomplete or biased model structure^91^. Model structure uncertainty can be examined through the use of multiple simulation tools or the selection of different model structures within the same modelling tool^92,93^. Additionally, the HSPF model needs improvement to include the effects of CO2 concentration on hydrological and water quality processes to truly reflect the effects of climate change on watershed systems. Although the uncertainties in HSPF model were not systematically evaluated here, it could be a part of future research.

Without considering all the factors discussed above, simulation results may not be realistic in some cases. Further process-specific research on internal processes would be beneficial to reduce uncertainties. However, the results of this study are useful to identify potential hydrological and water quality responses to different rainfall patterns and ongoing climate change. The findings of this study guide a robust adaptive management system to maintain the sustainability of water resources in the face of long dry spells and extreme precipitation.

## Conclusions

This study used the HSPF model to assess the potential impacts of rainfall patterns and droughts on watershed hydrology and NPS pollution, based on extreme climate projections obtained from five GCMs. Substantial increases in air temperature, precipitation intensity, frequency of heavy rains and rainstorms, and annual maximum duration of drought were predicted in future climate scenarios. In addition, the interacting factors affecting hydrology and water quality in response to rainfall patterns and drought conditions were identified. Precipitation, as a driving factor, played a major role in water balance and determined the amount of runoff. This caused serious soil erosion, nutrient losses, and outputs of fecal coliform by the interactive effects of raindrop and surface runoff. Hydrology and water quality were particularly vulnerable to short-term transient heavy rain and rainstorm events after a prolonged drought, with the comprehensive effects of antecedent soil water, antecedent drought duration, current precipitation amount and intensity, and antecedent precipitation characteristics. The results of this study can help to reveal the complex interactions of the various processes conditioning the water balance and linking agricultural NPS pollution and can guide a robust adaptive management system for future drinking water supply. Further research will focus on incorporating BMPs into adaptation strategies for climate extremes based on evaluating BMP performance in future climate change scenarios.

## Supplementary Information

Supplementary Information.

## Data Availability

The data that support the findings of this study are shown in the figures and tables of this paper and are available from the corresponding author upon reasonable request.
