# Supplementary material for: Synergistic effect of drought and rainfall events of different patterns on watershed systems
Source: Sci Rep. 2021 Sep 23;11:18957. doi: 10.1038/s41598-021-97574-z (PMC8460717; doi:10.1038/s41598-021-97574-z)
Supplement: Supplementary file 1 — Supplementary Information. [file 41598_2021_97574_MOESM1_ESM.docx]

Synergistic effect of drought and rainfall events of different patterns on watershed systems

Jiali Qiu^1^, Zhenyao Shen^1,*^, Guoyong Leng^2^, Guoyuan Wei^1^

^1^*State Key Laboratory of Water Environment Simulation, School of Environment, Beijing Normal University, Beijing 100875, PR China*

^2^*Key Laboratory of Water Cycle and Related Land Surface Processes, Institute of Geographic Sciences and Natural Resources Research, Chinese Academy of Sciences, Beijing 100101, China*

*Corresponding author: Zhenyao Shen

Tel/fax: +86 10 58804733

E-mail address: [zyshen@bnu.edu.cn](mailto:zyshen@bnu.edu.cn)

**Figures:**

Figure S1 Observed and GCM simulated daily precipitation (a) and temperature (b) during 1960—2005.

Figure S2 Projected changes in average monthly precipitation (a) and temperature (b) by 5 GCMs related to baseline climate.

**Tables:**

Table S1 Description of data used in the HSPF.

Table S2 Important parameters for pollutant transport simulation of HSPF.

Table S3 Monthly value of fecal coliform accumulation (MON- ACQOP).

Table S4 Monthly value of fecal coliform limit storage (MON- SQOLIM).

Table S5 Simulation results for different pollutants.

Table S6 Description of general circulation models (GCMs).

Table S7 Environmental Quality Standards for Surface Water (GB3838-2002) (unit: mg/L).





Figure S1 Observed and GCM simulated daily precipitation (a) and temperature (b) during 1960—2005. Graphs were created using OriginPro software version 9.0 (https://www.originlab.com/).





Figure S2 Projected changes in average monthly precipitation (a) and temperature (b) by 5 GCMs related to baseline climate. Graphs were created using OriginPro software version 9.0 (https://www.originlab.com/).

Table S1 Description of data used in the HSPF.

| Data type | Scale | Data description | Source |
| --- | --- | --- | --- |
| Digital Elevation Model | 1:50,000 | Elevation, overland and channel slopes and lengths | ASTER Global Digital Elevation Model downloaded from (<http://gdem.ersdac.jspacesystems.or.jp/>) |
| Land use | 1:10,000 | Land use classifications | The Present Land-use Map drawn by Forestry Bureau of Miyun County |
| Soil properties | 1:10,000 | Soil physical and chemical properties | Professional investigation report on demonstration area of water resource protection forest of Miyun Reservoir in Beijing City |
| Weather data | Hourly data | Precipitation, maximum and minimum air temperature, wind speed, cloud cover, relative humidity, potential evapotranspiration, and solar radiation | Local weather station and downscaled GCM projections |
| Hydrology and water quality | Hourly data | Flow discharge, concentrations of sediment, nutrients and bacteria at catchment outlet | Local environmental monitoring station |
| Social economical data |  | Population, livestock rearing, agricultural practices, fertilizer application | Field investigation and statistics yearbook |

Table S2 Important parameters for pollutant transport simulation of HSPF.

| Category | Name | Definition | Units | Current value |
| --- | --- | --- | --- | --- |
| Pervious Land Accumulation and Removal of Sediment (SEDMNT) Parameters | SMPF | Manegement Practice (P) factor from USLE | none | 0.9 |
|  | KRER | Coefficient in the soil detachment equation | complex | 0.325 |
|  | JRER | Exponent in the soil detachment equation | none | 2 |
|  | AFFIX | Daily reduction in detached sediment | 1/d | 0.002 (Forest)  0.011 (Urban)  0.015 (Agriculture) |
|  | COVER | Fraction land surface protected from rainfall | none | 0.97 (Forest)  0.90~0.95 (Urban)  0.50~0.90(Agriculture) |
|  | NVSI | Atmospheric additions to sediment storage | lb/ac.d | 1.5 (Forest)  1.2 (urban)  2.6 (agriculture) |
|  | KSER | Coefficient in the sediment washoff equation | complex | 0.26 (Forest)  0.15 (urban)  0.9 (agriculture) |
|  | JSER | Exponent in the sediment washoff equation | none | 2.2 |
|  | KGER | Coefficient in soil matrix scour equation | complex | 0 |
|  | JGER | Exponent in soil matrix scour equation | none | 1 |
| Instream Sediment Transport (SEDTRN) Parameters | KSAND | Coefficient in sandload power function formula | complex | 0.25 |
|  | EXPSND | Exponent in sandload power function formula | complex | 3.2 |
|  | TAUCD | Critical bed shear stress for deposition | lb/ft2 | 0.02~0.06 |
|  | TAUCS | Critical bed shear stress for scour | lb/ft2 | 0.07~0.12 |
|  | M | Erodibility coefficient | lb/ft2.d | 0.01 |
| Quality Constituents Using Simple Relationships - PQUAL  Wash-off of Quality Constituents Using Simple Relationships - IQUAL | SQO | Initial storage of QUALOF on the surface of the PERVIOUS LAND SEGMENT (PLS) and IMPERVIOUS LAND SEGMENT (ILS) | qty/ac | TAM: 0.03 (Forest), 0.455 (Urban), 0.875 (Agriculture)  NO3: 0.25 (Forest), 0.52 (Urban), 1.05 (Agriculture)  ORTHO P: 0.005(Forest), 0.02 (Urban), 0.024 (Agriculture)  FCOL: 1000 (Forest) , 6.2E+05 (Urban), 1E+08(Agriculture) |
|  | ACQOP | Rate of accumulation of QUALOF (on the surface) | qty/ac.d | TAM: 0.03  NO3: 0.08  ORTHO P: 0.012  FCOL: 5.24E+08 (Forest), 2.52E+09 (Urban), 1.81E+10 (Agriculture) |
|  | SQOLIM | Maximum storage of QUALOF | qty/ac | TAM: 0.04  NO3: 0.13  ORTHO P: 0.015  FCOL: 1.62E+09 (Forest), 4.80E+09 (Urban), 2.72E+10 (Agriculture) |
|  | WSQOP | Rate of surface runoff which will remove 90 percent of stored QUALOF per hour | in/hr | TAM: 0.5  NO3: 0.52  ORTHO P: 0.5  FCOL: 0.46 |
| Nitrogen Behavior - NITR | KDSAM | Ammonium desorption (only relevant if FORAFG = 0) | /day | 0.09 |
|  | KADAM | Ammonium adsorption (only relevant if FORAFG = 0) | /day | 0.03 |
|  | KAM | Organic N ammonification | /day | 0.04 |
|  | KNI | Nitrification | /day | 0.082 |
| Primary Inorganic Nitrogen Balances - NUTRX | KTAM20 | Nitrification rates of ammonia at 20 degrees C | /hr | 0.016 |
|  | KNO320 | Nitrate denitrification rate at 20 degrees C | /hr | 0.051 |
|  | KNO220 | Nitrification rates of nitrite at 20 degrees C | /hr | 0.002 |
| Phosphorous Behavior - PHOS | KDSP | Phosphate desorption (only used if FORPFG=0 in Table-type PHOS-FLAGS) | /day | 0.0012 |
|  | KADP | KADP Phosphate adsorption (only used if FORPFG=0 in Table-type PHOS-FLAGS) | /day | 0.001 |
|  | KIMP | Phosphate immobilization | /day | 0.0015 |
|  | KMP | Organic P mineralization | /day | 0.046 |
| Generalized Quality Constituent - GQUAL (for FCOL) | FSTDEC | First-order decay rate for qual | /day | FCOL: 0.52 |
|  | THFST | Temperature correction coefficient for first-order decay of qual | none | FCOL: 1.14 |

Table S3 Monthly value of fecal coliform accumulation (MON- ACQOP).

|  | Jan | Feb | Mar | Apr | May | Jun | Jul | Aug | Sep | Oct | Nov | Dec |
| --- | --- | --- | --- | --- | --- | --- | --- | --- | --- | --- | --- | --- |
| Agriculture | 1.13E+10 | 1.13E+10 | 1.13E+10 | 1.81E+10 | 1.81E+10 | 1.81E+10 | 1.13E+10 | 1.13E+10 | 1.13E+10 | 1.13E+10 | 1.13E+10 | 1.13E+10 |
| Forest | 3.80E+08 | 3.80E+08 | 3.80E+08 | 5.22E+08 | 5.24E+08 | 5.24E+08 | 5.24E+08 | 5.24E+08 | 5.24E+08 | 5.24E+08 | 3.80E+08 | 3.80E+08 |
| Urban | 2.52E+09 | 2.52E+09 | 2.52E+09 | 2.52E+09 | 2.52E+09 | 2.52E+09 | 2.52E+09 | 2.52E+09 | 2.52E+09 | 2.52E+09 | 2.52E+09 | 2.52E+09 |

Table S4 Monthly value of fecal coliform limit storage (MON- SQOLIM).

|  | Jan | Feb | Mar | Apr | May | Jun | Jul | Aug | Sep | Oct | Nov | Dec |
| --- | --- | --- | --- | --- | --- | --- | --- | --- | --- | --- | --- | --- |
| Agriculture | 1.62E+09 | 1.62E+09 | 1.62E+09 | 2.72E+09 | 2.72E+10 | 2.72E+10 | 1.71E+10 | 1.71E+10 | 2.64E+10 | 2.77E+10 | 5.25E+09 | 4.40E+09 |
| Forest | 1.62E+09 | 1.62E+09 | 1.62E+09 | 1.62E+09 | 1.62E+09 | 1.62E+09 | 1.62E+09 | 1.62E+09 | 1.62E+09 | 1.62E+09 | 1.51E+09 | 1.51E+09 |
| Urban | 4.80E+09 | 4.80E+09 | 4.80E+09 | 4.80E+09 | 4.80E+09 | 4.80E+09 | 4.80E+09 | 4.80E+09 | 4.80E+09 | 4.80E+09 | 4.80E+09 | 4.80E+09 |

Table S5 Simulation results for different pollutants.

| Variables | Flow | | Sediment | | TN | | NO3-N | | TAM | | TP | | FCOL | |
| --- | --- | --- | --- | --- | --- | --- | --- | --- | --- | --- | --- | --- | --- | --- |
|  | *R2* | *Ens* | *R2* | *Ens* | *R2* | *Ens* | *R2* | *Ens* | *R2* | *Ens* | *R2* | *Ens* | *R2* | *Ens* |
| Daily flow | 0.957 | 0.891 | - | - | - | - | - | - | - | - | - | - | - | -- |
| 2014-06-16 | 0.815 | 0.747 | 0.877 | 0.544 | 0.794 | 0.503 | 0.773 | 0.221 | 0.818 | 0.572 | 0.736 | 0.521 | 0.502 | 0.369 |
| 2014-07-01 | 0.868 | 0.779 | 0.862 | 0.746 | 0.846 | 0.737 | 0.88 | 0.835 | 0.659 | 0.633 | 0.781 | 0.551 | 0.532 | 0.463 |
| 2014-07-21 | 0.695 | 0.447 | 0.896 | 0.557 | 0.0222 | -5.422 | 0.691 | -0.927 | 0.647 | 0.407 | 0.296 | -1.196 | 0.124 | -0.275 |
| 2014-08-23 | 0.744 | 0.591 | 0.895 | 0.482 | 0.685 | 0.478 | 0.685 | 0.514 | 0.789 | 0.557 | 0.818 | 0.761 | 0.433 | 0.315 |
| 2014-08-28 | 0.827 | 0.775 | 0.904 | 0.830 | 0.833 | 0.766 | - | - | - | - | 0.835 | 0.698 | 0.217 | -1.451 |
| 2014-09-01 | 0.941 | 0.829 | 0.850 | 0.507 | 0.649 | 0.518 | - | - | - | - | 0.755 | 0.579 | 0.337 | 0.201 |
| 2015-07-19 | 0.930 | 0.872 | 0.911 | 0.557 | 0.653 | 0.422 | - | - | - | - | 0.721 | 0.610 | 0.353 | 0.138 |
| 2015-07-20 | 0.895 | 0.841 | 0.920 | 0.763 | 0.854 | 0.761 | - | -- | - | -- | 0.555 | 0.391 | 0.610 | -3.292 |

Table S6 Description of general circulation models (GCMs).

| Model name | Expansion | Institute acronyms | Institute full name |
| --- | --- | --- | --- |
| GFDL-ESM2M | Geophysical Fluid Dynamics Laboratory Earth System Model with Modular Ocean Model 4 (MOM4) component (ESM2M) | NOAA GFDL | NOAA Geophysical Fluid Dynamics Laboratory |
| HadGEM2-ES | Hadley Centre Global Environment Model, version 2 (Earth System) | MOHC (additional realizations by INPE) | Met Office Hadley Centre and Instituto Nacional de Pesquisas Espaciais |
| IPSL-CM5A-LR | L’Institut Pierre-Simon Laplace Coupled Model, version 5, coupled with Nucleus for European Modelling of the Ocean (NEMO), low resolution | IPSL | L’Institut Pierre-Simon Laplace |
| MIROC-ESM-CHEM | Model for Interdisciplinary Research on Climate, Earth System Model, Chemistry Coupled | MIROC | Japan Agency for Marine-Earth Science and Technology, Atmosphere and Ocean Research Institute (The University of Tokyo), and National Institute for Environmental Studies |
| NorESM1-M | Norwegian Earth System Model, version 1 (intermediate resolution) | NCC | Norwegian Climate Centre |

Table S7 Environmental Quality Standards for Surface Water (GB3838-2002) (unit: mg/L).

| Variable | Standard limits | | | | |
| --- | --- | --- | --- | --- | --- |
|  | Class I | Class II | Class III | Class IV | Class V |
| Total nitrogen | 0.2 | 0.5 | 1.0 | 1.5 | 2.0 |
| Total phosphorous | 0.02  0.01 (for reservoir and lake) | 0.1  0.025 (for reservoir and lake) | 0.2  0.05 (for reservoir and lake) | 0.3  0.1 (for reservoir and lake) | 0.4  0.2 (for reservoir and lake) |
| Fecal coliforms (UFC/L) | 200 | 2000 | 10000 | 20000 | 40000 |
